# Attitudes toward genetic testing, family planning and preimplantation genetic testing in families with a germline *CDKN2A* pathogenic variant

**DOI:** 10.1007/s10689-024-00401-3

**Published:** 2024-06-01

**Authors:** A. M. Onnekink, D. C.F. Klatte, J. E. van Hooft, S. H. van den Berg, S. M.S. van der Zwaan, R. van Doorn, S. C.H. Hinnen, T. P. Potjer, E. M.A. Bleiker, M. E. van Leerdam

**Affiliations:** 1 Department of Gastroenterology and Hepatology, Leiden University Medical Center, Albinusdreef 2, Leiden, 2333 ZA The Netherlands; 2 Department of Gastroenterology and Hepatology, Mayo Clinic, Jacksonville, FL USA; 3 Department of Dermatology, Leiden University Medical Center, Leiden, the Netherlands; 4 Department of Psycho-Oncology, Leiden University Medical Center, Leiden, the Netherlands; 5 Department of Medical Psychology, Spaarne Gasthuis, Haarlem, the Netherlands; 6 Department of Clinical Genetics, Leiden University Medical Center, Leiden, The Netherlands; 7 Division of Psychosocial Research and Epidemiology, Netherlands Cancer Institute, Amsterdam, The Netherlands; 8 Department of Clinical Genetics, The Netherlands Cancer Institute, Amsterdam, The Netherlands; 9 Department of Gastrointestinal Oncology, The Netherlands Cancer Institute, Amsterdam, the Netherlands

## Abstract

Individuals with a germline *CDKN2A* pathogenic variant (PV) have a highly increased life time risk of melanoma and pancreatic cancer. This cross-sectional study assessed the attitudes among toward genetic testing, family planning, and preimplantation genetic testing (PGT) in confirmed *CDKN2A* PV carriers and individuals with a 50% risk of the PV (at-risk carriers) using of a one-time questionnaire.

A total of 537 individuals were screened for eligibility, of whom 208 of 366 (57%) confirmed carriers (56% female, median age 54 years [IQR 46–63]) and 39 of 171 (23%) at-risk carriers (59% female, median age of 26 years [IQR 22–32]) participated in the study. Primary motivations for genetic testing were to gain control over their personal and children’s cancer risk, as well as increasing cancer surveillance practices. In contrast, concerns about obtaining a mortgage and life insurance were frequently cited as reasons for postponing genetic testing. Family planning decisions remained largely unaffected in both confirmed and at-risk carriers; however, the majority of confirmed carriers were still unaware of their familial or personal cancer risk when starting a family. More than 60% of the participants were unfamiliar with PGT and only a minority (19% of confirmed carriers and 10% of at-risk carriers) would be open to considering PGT as a reproductive option. This study found different attitudes toward genetic testing, family planning, and PGT among individuals affected by the *CDKN2A* PV. Understanding these different attitudes can help clinicians to address the complexities surrounding these issues, especially for younger individuals facing difficult decisions about the timing of genetic testing, family planning, and the potential use of assisted reproductive options.

**Supplementary Information:**

The online version contains supplementary material available at 10.1007/s10689-024-00401-3.

## Introduction

Hereditary melanoma, an autosomal dominant inherited cancer syndrome, is predominantly caused by a germline pathogenic variant (PV) in the *CDKN2A* gene. The Dutch founder *p16*-Leiden variant, an inactivating 19-base pair deletion in *CDKN2A* (c.225_243del19), is the most common PV in the Netherlands. Individuals with a germline *CDKN2A* PV have an increased lifetime risk of up to 70% for melanoma and up to 20% for pancreatic cancer, and are recommended to undergo skin and pancreatic cancer surveillance [1–5]. Skin surveillance can be initiated at age 12 and is offered every six months to confirmed carriers and annually to their first-degree relatives, who have a 50% chance of having inhered the PV (henceforth referred to as at-risk carriers) [5]. Pancreatic cancer surveillance, however, is only offered to those with a PV confirmed by genetic testing. The surveillance program consists of annual magnetic resonance imaging (MRI) and, if necessary, endoscopic ultrasound (EUS), and starts at age 40 years or 10 years before the youngest familial onset of pancreatic cancer [1, 3].

Our study group previously conducted a focus group study with confirmed and at-risk carriers and found that the majority of participants expressed a preference for postponing testing until age 40 to become eligible for pancreatic cancer surveillance [6]. Another important motive to pursue genetic testing was to obtain certainty about their own and children’s cancer risk. Notably, most confirmed carriers in our focus group study already had children prior to genetic testing. The extent to which their PV carrier status played a role in family planning decisions varied among participants, with some reporting no impact on family planning, while others reported a shift toward having fewer or no children [6]. Previous studies of individuals diagnosed with rare hereditary cancer syndromes such as Peutz-Jeghers syndrome (PJS) and familial adenomatous polyposis (FAP) found that 29% and 37% of participants, respectively, had adjusted their reproductive choices following their diagnosis (i.e., decided to have fewer or no children) [7, 8]. Thus, given that confirmed carriers have a 50% chance of passing on the cancer predisposition to their children, early awareness of one’s personal cancer risk can significantly influence family planning decisions.

Preimplantation genetic testing (PGT) is an available yet little-used reproductive option in the family planning decision-making process for individuals diagnosed with hereditary cancer syndromes in the Netherlands [9]. Individuals at risk for hereditary cancer have limited knowledge of preimplantation genetic testing, with only 35% having heard of it, as shown by a previous meta-analysis [10].

Currently, little is known about the attitudes of individuals with the *CDKN2A* PV toward genetic testing, family planning, and PGT. Therefore, we conducted a quantitative survey to assess these attitudes among confirmed and at-risk carriers of *CDKN2A* PV. In addition, we investigated determinants associated with a positive attitude toward PGT.

## Methods

### Study population

Individuals with a confirmed germline *CDKN2A* PV by genetic testing (i.e., confirmed carriers) or those with a 50% risk of carrying the PV (i.e., at-risk carriers) participating in the skin and pancreatic cancer surveillance programs Leiden University Medical Center (LUMC), the Netherlands, were eligible for the study. Skin surveillance is offered every 6 months to confirmed carriers and annually to at-risk carriers, starting at the age of 12, while pancreatic cancer surveillance is only offered to confirmed carriers at the age of 40 or 10 years before the youngest familial onset. Details of the LUMC cancer surveillance programs have been described elsewhere [1, 5]. Other eligibility criteria for the study included a minimum age of 18 years and proficiency in Dutch language. Confirmed carriers, having already undergone testing, were likely to have experienced various consequences of a positive test result. Conversely, at-risk carriers, who were typically younger, were expected to be facing upcoming decisions regarding genetic testing, family planning, and possibly the use of PGT. Due to inherent group differences, this study opted to describe the attitudes of confirmed and at-risk carriers separately, deeming direct comparisons less meaningful Participants were included between February 2023 and July 2023. The study was approved by the Medical Ethical Committee of the LUMC (MEC P22.084).

### Procedures

Eligible individuals were contacted by letter and invited to participate in the study by completing a one-time questionnaire on genetic testing, family planning, and PGT. After 6 weeks, non-responders were reminded of the study by letter or in person during a pancreatic surveillance follow-up visit by the study team. After providing written informed consent, individuals were sent a digital questionnaire or a paper version upon request. The questionnaire is provided as supplementary material. A reminder to complete the questionnaire was sent if individuals had not responded within 6 weeks.

### Measures

Sociodemographic and clinical variables were obtained by self-report questions. Sociodemographic variables were sex, age, having a partner, having children, educational level, and employment status. Educational level was categorized as high (i.e., higher secondary school, college or university) or low (i.e., only primary school, lower secondary school, lower or intermediate vocational school). Employment covered both paid and unpaid work, such as volunteer work. Clinical variables included genetic status (i.e., confirmation of a germline *CDKN2A* PV), as well as personal and family history of cancer, the latter defined by the prevalence of cancer in a first-degree relative.

The outcomes of interest included the attitude toward genetic testing, family planning, and PGT. Study-specific questions regarding these topics were based on previous literature [7, 8, 11].

Attitudes toward genetic testing were assessed with questions about the willingness to undergo genetic testing among at-risk carriers, reasons for genetic testing among confirmed carriers and at-risk carriers who were open to genetic testing, and reasons for not undergoing genetic testing among at-risk carriers who were hesitant or reluctant to undergo genetic testing. Participants could give a maximum of three reasons why they would or would not consider genetic testing. In addition, participants with biological children were asked whether they would recommend genetic testing to their children and what age they would consider most appropriate for testing.

Attitudes toward family planning included questions about the desire to have (more) children and assessed various attitudes about whether PV carrier status affects family planning. Furthermore, both confirmed and at-risk carriers were asked about their prior knowledge of PGT and, after a brief introduction to PGT, whether they would receive counseling about PGT and be willing to consider using this reproductive method.

The questionnaire was pilot tested for readability and comprehension in a small random selection of individuals with hereditary cancer predisposition syndromes other than hereditary melanoma and individuals from the general population.

### Statistical analysis

Descriptive statistics were used to characterize the study population and expressed as percentages, means with standard deviations (SD) or medians with interquartile range (IQR), depending on data distribution. Results were stratified by genetic status, i.e., those with a confirmed PV carrier status and those at risk of the PV (50% risk). A logistic regression model was used to assess the association of demographic and clinical variables on a favorable attitude toward PGT. Variables with a p-value < 0.10 in the univariable analysis, as well as sex and age, were entered into the multivariate regression model. Statistical analyses with a two-sided p-value < 0.05 were considered statistically significant. All statistical analyses were performed using SPSS 26.0 (IBM Corporation, Armonk, New York, USA).

## Results

### Participant characteristics

**Fig. 1:**
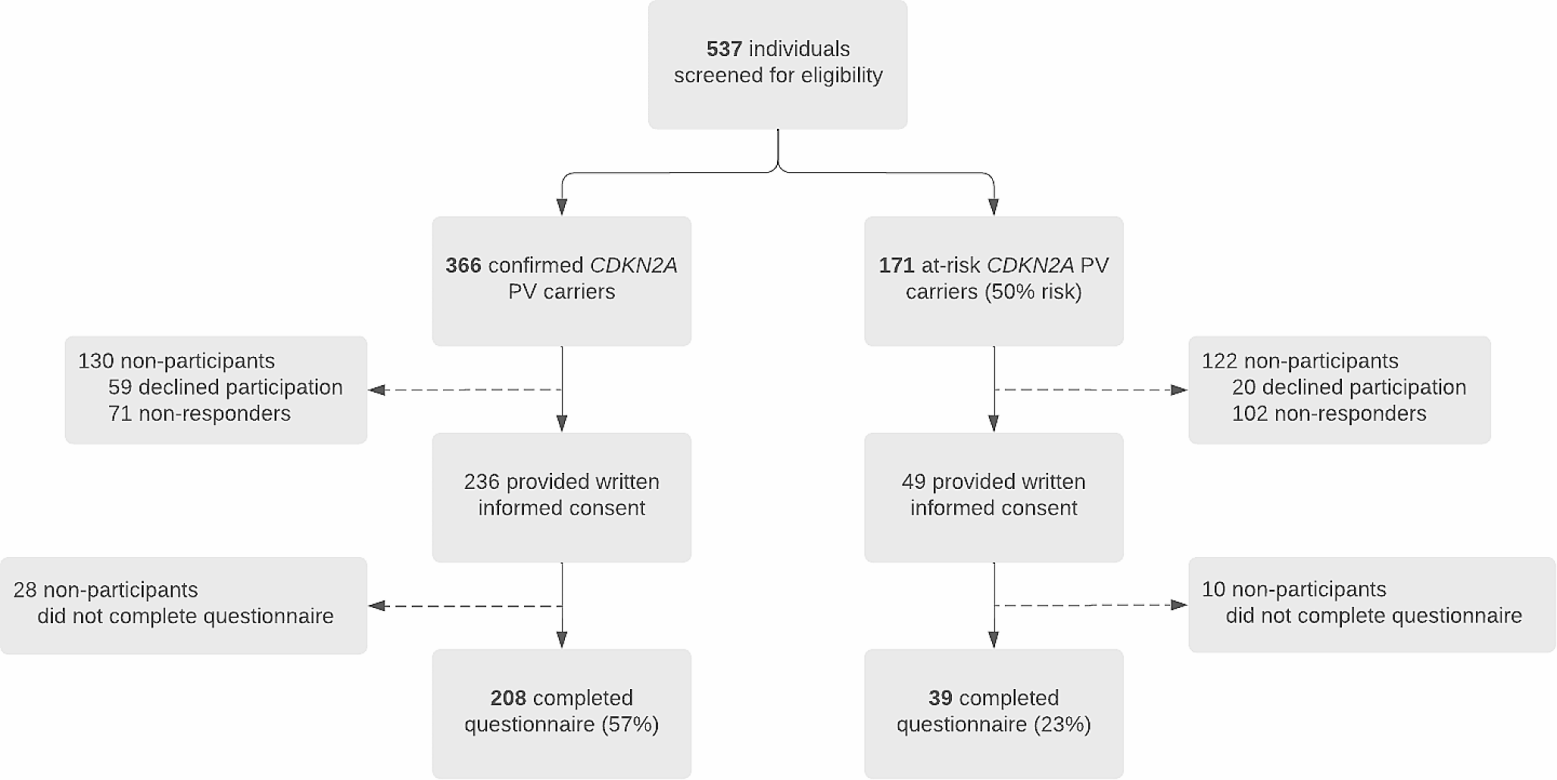
**Flow diagram of study** Confirmed carriers had the PV confirmed by genetic testing, while at-risk carriers, who had a 50% risk of carrying the PV, had not (yet) undergone genetic testing

A total of 537 confirmed *CDKN2A* PV carriers and at-risk carriers under skin and pancreatic cancer surveillance were invited for this study, of whom 247 participated. The questionnaire was completed by 208 of 366 (57%) confirmed carriers and 39 of 171 (23%) at-risk carriers (Fig. 1). Study participants with a confirmed PV were older compared with non-participants (54 years [IQR 46–63] vs. 51 years [IQR 39–56], respectively, *P* < 0.01; Table 1). Among the at-risk carriers, no differences in terms of sex and age were observed between study participants and non-participants.

**Table 1:** Characteristics of study participants and non-participants

	Confirmed CDKN2A PV carriers			At-risk CDKN2A PV carriers		
	Study participants **(** ***n*** **= 208)**	Non-participants^a^ **(** ***n*** **= 158)**	*P*-value	Study participants **(** ***n*** **= 39)**	Non-participants^a^ **(** ***n*** **= 132)**	*P*-value
Sex, n (%)						
Female	116 (56%)	95 (60%)	0.40	23 (59%)	54 (41%)	0.07
Male	92 (44%)	63 (40%)		16 (41%)	78 (59%)	
Age, median (IQR)	54 (46–63)	51 (39–56)	**< 0.01**	26 (22–32)	25 (21–33)	0.53

PV; pathogenic variant, IQR; interquartile range

^a^ All study participants completed the questionnaire. Non-participants were non-responders and those who declined study participation or had not completed the questionnaire

Study participants’ characteristics are summarized in Table 2. Confirmed carriers, with a median age of 54 years (IQR 46–63), predominantly (89%) participated in both skin and pancreatic cancer surveillance programs. At-risk carriers, with a median age of 26 years (IQR 22–32), participated in skin surveillance only, as they were not yet eligible for pancreatic cancer surveillance. Most of the confirmed carriers (89%) had children, compared with 18% of at-risk carriers. In addition, confirmed carriers (51%) had a higher prevalence of personal history of cancer, especially melanoma, than at-risk carriers (13%).

**Table 2:** Baseline characteristics of confirmed and at-risk carriers

	Confirmed carriers (*n* = 208)	At-risk carriers (*n* = 39)
Sex		
Female	116 (56%)	23 (59%)
Male	92 (44%)	16 (41%)
Age, median (IQR)	54 (46–63)	26 (22–32)
Cancer surveillance		
Skin surveillance only	23 (11%)	39 (100%)
Skin and pancreatic surveillance	185 (89%)	0
Having a partner	179 (86%)	29 (74%)
Having one or more children	185 (89%)	7 (18%)
High educational level	74 (36%)	20 (51%)
Employment	168 (81%)	33 (85%)
Personal history of malignancy Melanoma Pancreatic cancer Other	121 (58%) 106 (51%) 7 (3%) 32 (15%)	7 (18%) 5 (13%) 0 (0%) 2 (5%)
Malignancy in family (FDR)		
Melanoma	154 (74%)	28 (74%)
Pancreatic cancer	71 (34%)	4 (11%) ^a^
Other	124 (60%)	11 (29%) ^a^

Data are presented as n (%) unless otherwise specified. Percentages may not sum to 100 because of rounding

PV; pathogenic variant, IQR; interquartile range, FDR; first-degree relative

^a^ 1 missing

### Attitudes toward genetic testing

Within the group of confirmed carriers, participants became aware of their potential *CDKN2A* PV carrier status at a median age of 36 years (IQR 27–48). Genetic testing was subsequently undertaken at a median age of 42 years (IQR 34–50) (Table 3). The most common motivations for individuals to undergo genetic testing were (i) to intensify skin surveillance or initiate pancreatic cancer surveillance (52%), (ii) to gain more certainty about their own cancer risk (41%), and (iii) for their children (33%). More than 80% of confirmed carriers would recommend genetic testing to their children (22% probably and 61% definitely). The majority (57%) suggested the ages between 18 and 35 as the most suitable for genetic testing, while 10% advised earlier testing (12–17 years) and 18% suggested later testing (36–45 years). The primary rationale for genetic testing between the ages of 18 and 35 was to ensure that at-risk carriers would be old enough to make informed decisions about their hereditary cancer predisposition and its implications, including the potential financial consequences and reproductive decisions upon confirmation of the *CDKN2A* PV. The age group of 18–35 years encompasses the childbearing years, and information about one’s carrier status can influence reproductive decision-making. Motivations for testing between the ages of 36 and 45 years were based on avoiding difficulties in obtaining a mortgage or life insurance, and becoming eligible for pancreatic cancer surveillance once the PV was confirmed.

**Table 3:** Attitudes toward genetic testing

	Confirmed carriers (*n* = 208)	At-risk carriers (*n* = 39)
Age of becoming aware of genetic predisposition, median (IQR)	36 (27–48)	14 (12–16)
Desire to undergo genetic testing?		
*Yes*	NA	18 (46%)
*Not sure*		18 (46%)
*No*		3 (8%)
Age at testing ^a^	42 (IQR 34–50)	NA
Reasons to undergo in genetic testing ^b, c^		
*… To become eligible for pancreatic cancer surveillance and/or intensify skin surveillance*	107 (52%)	10 (77%)
… *To gain more certainty about my risk of melanoma and pancreatic cancer*	84 (41%)	10 (77%)
*… For my children*	68 (33%)	2 (15%)
*… To feel a greater sense of control*	37 (18%)	3 (23%)
*… For family planning decisions*	7 (3%)	5 (38%)
… *General future planning (other than family planning, such as work or relationships)*	2 (1%)	1 (8%)
*… A healthcare professional has referred me*	24 (12%)	-
*… Requested by a family member*	13 (6%)	-
*… To contribute to scientific research*	22 (11%)	-
*… Other*	6 (3%)	-
Reasons not to engage in genetic testing ^b^		
*… I am still too young*		5 (24%)
*… I already undergo skin checks and therefore feel that genetic testing is not necessary*		11 (52%)
*… The genetic test result may have financial implications for obtaining a mortgage/insurance*		11 (52%)
*… I do not want the genetic test result to affect my daily life*		6 (29%)
*… The test will increase my fear of cancer*		3 (14%)
*… I do not want the test result to impact my family planning decisions*		3 (14%)
*… Testing was discouraged by my close friends or family members*		3 (14%)
*… I find it to be confronting to undergo testing*		2 (10%)
… *Other*		2 (10%)
Would you recommend your children to pursue genetic testing? ^d^		
*No*	4 (2%)	0 (0%)
*Maybe*	28 (16%)	5 (71%)
*Probably yes*	39 (22%)	0 (0%)
*Yes, definitely*	109 (61%)	2 (29%)
What age would you consider most appropriate for genetic testing? ^e^		
*12–17*	18 (10%)	0 (0%)
*18–25*	53 (29%)	1 (14%)
*26–35*	50 (28%)	1 (14%)
*36–45*	32 (18%)	2 (29%)
*Other*	27 (15%)	3 (43%)

PV, pathogenic variant; IQR, interquartile range; NA, not applicable

^a^ 5 confirmed carriers missing. ^b^ Participants were asked to fill in a maximum of 3 motivations

^c^ 3 confirmed carriers missing and 5 of 18 at-risk carriers missing who were willing to engage in genetic testing

^d^ Assessed among confirmed and at-risk carriers (*n* = 183*)* with biological children; 3 confirmed carriers missing

At-risk carriers had a median age of 26 years (IQR 22–32) and had learned of their 50% risk carrier status at a median age of 14 years (IQR 12–16). A total of 16 at-risk carriers (41%) had received genetic counseling by a clinical geneticist. 31% of at-risk carriers were concerned about being a PV carrier, 44% were somewhat concerned, and 26% were not at all concerned about their risk carrier status. 8% expressed that they did not want to undergo genetic testing, whereas the remaining at-risk carriers were not sure yet (46%) or preferred to undergo genetic testing at a later time (46%). The majority of those motivated to undergo genetic testing aimed to gain more certainty about their cancer risk (77%) and to intensify skin surveillance or initiate pancreatic cancer surveillance (77%). Family planning was cited as a motivation for genetic testing by 38% of at-risk carriers. Among those who were uncertain or unwilling to undergo genetic testing, the main concerns were the potential financial consequences of a positive result (52%), particularly with regard to mortgages and life insurance, and the belief that testing was unnecessary because at-risk carriers could participate in annual skin surveillance without testing (52%). Other reasons for declining or delaying genetic testing included concerns about the potential impact of testing results on their daily lives (29%) and feeling too young to undergo genetic testing (24%).

### Attitudes toward family planning

A total of 183 confirmed carriers (88%) had one or more biological children, and most had already fulfilled their desire to have children (80%) (Table 4). The majority of PV carriers started a family without knowing their carrier status, either because they were unaware of their family’s hereditary cancer predisposition (68%) or because they had not yet undergone genetic testing at the time (34%). Consequently, their family planning decisions were not influenced by their carrier status. Feelings of guilt were expressed by 35% of confirmed carriers towards their children regarding the possibility of transmitting the PV.

**Table 4:** Attitudes toward family planning

	Confirmed carriers (*n* = 208)	At-risk carriers (*n* = 39)
Having biological children	183 (88%)	7 (18%)
Desire to have children ^a^		
*Yes*	7 (3%)	26 (67%)
*No already fulfilled*	167 (80%)	5 (13%)
*No desire*	26 (13%)	4 (10%)
*Don’t know*	6 (3%)	4 (10%)
Did the PV influence family planning? ^a^		
*No*	182 (88%)	29 (74%)
*A bit*	10 (5%)	6 (15%)
*Yes*	14 (7%)	4 (10%)
Why did the PV not impact family planning? ^b^ *Because …*		
*… I did not know about the presence of the CDKN2A PV in my family when starting a family*	124 (68%)	3 (10%)
*… I had not yet undergone genetic testing (before having children)*	62 (34%)	8 (28%)
… *The CDKN2A PV does not necessarily have to result in serious health consequences*	22 (12%)	18 (62%)
*… I believe that the chances that I will get sick in the future are low*	2 (1%)	3 (10%)
*… I believe that the chances that my children will get sick in the future are low*	7 (4%)	6 (21%)
*… Of religious considerations*	2 (1%)	0 (0%)
*… Other*	21 (12%)	5 (17%)
Why did the PV impact family planning? ^c^ *Because …*		
*… I am/was afraid that my child could be a carrier*	20 (83%)	9 (90%)
*… I am/was ill or expect to become sick in the future*	5 (21%)	3 (30%)
*… I would find it difficult that one child could be a carrier while the other child is not*	4 (17%)	3 (30%)
*… My partner is/was afraid that our child could be a PV carrier*	2 (8%)	1 (10%)
*… Other*	4 (17%)	2 (20%)
Feelings of guilt toward children about possibility of passing on the hereditary predisposition ^d^		
*Yes*	22 (13%)	1 (14%)
*Quite a bit*	37 (22%)	0 (0%)
*A bit*	67 (40%)	3 (43%)
*No*	43 (25%)	3 (43%)

PV, pathogenic variant. ^a^ 2 confirmed carriers missing. ^b^ Multiple answers possible. Assessed among 182 confirmed carriers and 29 at-risk carriers whose family planning decisions were not influenced by their (risk) carrier status. ^c^ Multiple answers possible. Assessed among 24 confirmed carriers and 10 at-risk carriers who expressed that their (risk) carrier status had affected their family planning decisions to some degree. ^d^ 14 of 183 confirmed carriers missing

Among the at-risk carriers, 7 (18%) had biological children and 26 (67%) had an active desire to have children. The majority of at-risk carriers (74%) indicated that their family planning decision were not influenced in light of their risk carrier status. The main reasons for this were that 62% believed that a positive carrier status did not necessarily have serious health consequences, and 28% reported not being aware of their own cancer risk as they had not yet undergone genetic testing. Conversely, 9 out of 10 (90%) at-risk carriers who reported that their risk carrier status had affected their family planning, were concerned that their children might inherit PV.

### Attitudes toward PGT

**Fig. 2:**
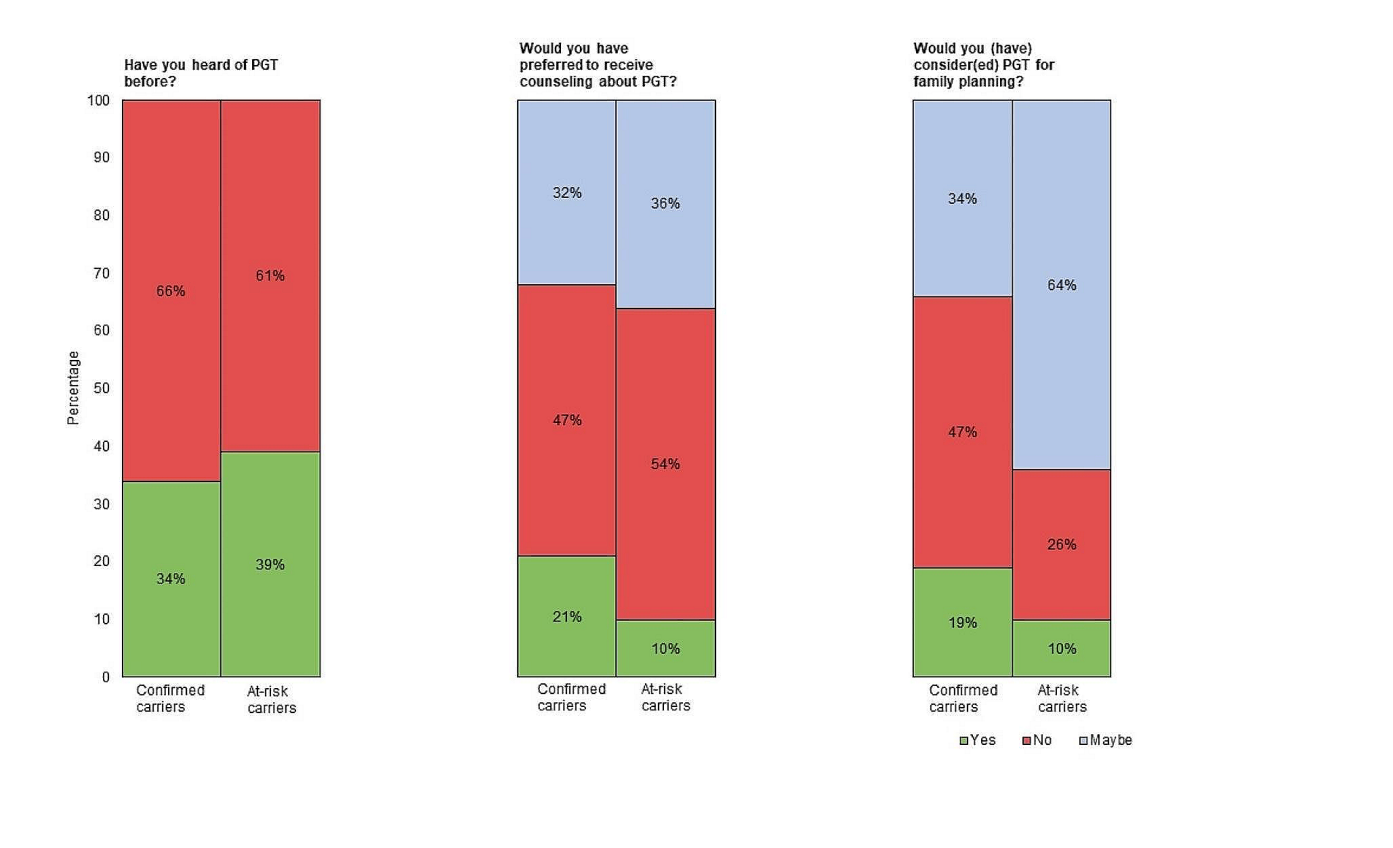
PV, pathogenic variant. ^a^ 6 confirmed carriers missing. ^b^ 9 confirmed carriers missing

More than 60% of both confirmed and at-risk carriers had not heard of, or did not recall having heard of PGT before (Fig. 2, Table S1). Interest in PGT counseling was expressed by 21% of confirmed carriers and by 10% of at-risk carriers. Among confirmed carriers, 19% would have been willing to consider PGT as a reproductive option, while 33% were hesitant and 47% would not consider its use. At-risk carriers expressed different attitudes toward PGT, as 10% were willing to consider PGT, 64% were hesitant, and 26% were reluctant. Among 35 at-risk carriers identified as being of childbearing age (≤ 40 years), 14 (40%) had received genetic counseling about PGT from a clinical geneticist.

Univariable analysis showed that individuals with a lower age, without a confirmed PV, and a higher education level were more inclined to have a positive attitude toward PGT (Table 5). In the multivariable analysis, after accounting for sex and age, being an at-risk PV carrier remained significantly associated with a positive attitude toward PGT (OR for confirmed PV 0.35, 95% CI 0.14 to 0.89).

**Table 5:** Regression analysis of determinants associated with a positive attitude toward PGT

Positive attitude toward the use of PGT	All study participants			
	Univariate analysis		Multivariable analysis	
	OR (95% CI)	*P*-value	OR (95% CI)	*P*-value
Sex (male/female)	0.80 (0.47 to 1.35)	0.40		NS
Age (continuous)	0.97 (0.96 to 0.99)	**< 0.01**		NS
Confirmed PV carrier status (yes/no)	0.28 (0.14 to 0.57)	**< 0.01**	0.35 (0.14 to 0.89)	**0.03**
Education (high/low)	1.69 (0.99 to 2.88)	0.06		NS
Children (yes/no)	0.72 (0.39 to 1.32)	0.29		
Personal history of cancer (yes/no)	0.64 (0.38 to 1.09)	0.10		
FDR with melanoma or pancreatic cancer (yes/no)	0.89 (0.39 to 2.01)	0.78		

PV, pathogenic variant; FDR, first-degree relative; NS, not significant. P-values < 0.10 were entered in multivariable logistic regression analysis. P-values < 0.05 are denoted in bold

## Discussion

This is the first large-scale quantitative study to report a variety of attitudes toward genetic testing, family planning, and PGT in families affected by the *CDKN2A* PV.

### Attitudes toward genetic testing

An increasing number of families affected by a *CDKN2A* PV have been identified over the past two decades. In the current study, most confirmed carriers became aware of their potential carrier status after they had children (median age of 36 years) and subsequently underwent genetic testing after the age of 40. By then, confirmed carriers were eligible to participate in pancreatic cancer surveillance, which was reported to be an important motivation for seeking genetic testing. In addition, the skin surveillance interval was shortened to every six months following confirmation of *CDKN2A* PV. Both skin and pancreatic cancer surveillance programs have been shown to detect tumors at more favorable prognostic stages, leading to improved survival outcomes [1, 3, 12, 13].

Other important motivations for genetic testing were the desire to gain knowledge about their own and children’s cancer risk. These motivations were in line with a previous cohort study of Australian family members with hereditary melanoma due to a *CDKN2A* PV [14]. However, while 67% of the at-risk carriers in this Australian cohort were willing to undergo genetic testing, only 21% ultimately did so. This low testing rate may be due to the understanding that a negative test result did not eliminate the importance of skin protection and skin self-examination. Moreover, there was little evidence of an elevated lifetime risk of pancreatic cancer within these Australian families, potentially further diminishing the necessity for genetic testing. Although the specific *CDKN2A* PV was not disclosed in this Australian study, most PVs described in the literature affect the p16INK4a protein, generally correlating with a 15–20% lifetime risk of pancreatic cancer [15].

In our study cohort, the association between the *p16*-Leiden variant and pancreatic cancer has been well established [1, 16]. Nearly half (46%) of at-risk carriers had a positive attitude towards genetic testing, generally driven by a desire to learn more about their personal cancer risk and to improve cancer screening practices. However, considering that the typical age of onset of pancreatic cancer in this population is around 55 years, and pancreatic cancer surveillance requires a minimum age of 40 years, it is plausible that at-risk carriers may postpone genetic testing until they reach this surveillance age threshold [17]. Interestingly, a contrasting perspective was expressed by confirmed carriers, with the majority recommending earlier testing (18–35 years) for their children. This age preference for testing was motivated by a desire to equip their children with the knowledge necessary to make autonomous decisions about genetic testing, family planning and potential financial implications of a positive test result. Our previous focus group study found that most at-risk carriers primarily rely on information and experiences shared by family members, with limited use of formal genetic counselling from a clinical geneticist [6]. Approximately half of the at-risk carriers in the current study showed hesitancy or reluctance toward genetic testing and expressed concern about the financial consequences of a positive test result, particularly with regard to obtaining a mortgage and life insurance. However, in the Netherlands, life insurance is not mandatory for obtaining a mortgage, and questions about hereditary cancer predisposition for life insurance are limited below €328,131 (legal question limit as of July 1, 2023) [18, 19]. Moreover, insurance companies often use even higher thresholds, implying that a positive genetic test may have less financial impact than perceived, as confirmed carriers generally qualify for standard disability or life insurance coverage under normal terms and conditions. However, it should be noted that the financial implications can be different for individuals with a personal history of cancer or if the insured amount exceeds this legal limit. Genetic counseling offers a tailored approach to these complexities, and we encourage at-risk carriers to seek such advice.

### Attitudes toward family planning and PGT

The vast majority of confirmed carriers (88%) made their family planning decisions independently of their hereditary cancer predisposition, either because they were unaware of their family’s genetic risk or because they had not yet undergone genetic testing at the time of starting a family. Some participants expressed a sense of relief in not knowing their genetic risk, and thus avoiding the burden of contemplating family planning decisions [6]. Interestingly, among at-risk carriers, all of whom were aware of their PV carrier status, three-quarters reported that this knowledge did not affect their family planning decisions. This consideration was primarily motivated by the belief that a positive PV carrier status does not necessarily translate into serious health consequences. This was supported by previous results from a Norwegian cohort study of families with a *CDKN2A* PV [20]. The Norwegian study identified seven distinct *CDKN2A* PVs across 18 different families, all of which were associated with a high risk of melanoma and a potential risk of pancreatic cancer. However, the uptake of genetic counseling and testing was relatively low compared with a similar cohort of *BRCA1* families. This discrepancy may be explained by differences in perceived disease severity, as most *CDKN2A* PV carriers had survived melanoma, in contrast to the *BRCA1* cohort, where a large percentage of affected relatives in the *BRCA1* cohort had died of their disease [21]. Other previous qualitative studies of hereditary breast and ovarian cancer (HBOC) identified the familial domain as an important area of concern. Carriers expressed concerns about their children’s genetic status, fearing that their children may face similar issues, including cancer diagnoses, witnessing family loss, or encountering difficulties in finding a partner or making reproductive decisions [22–25].

PGT presents a potential option for individuals with a confirmed *CDKN2A* PV with concerns regarding family planning. This reproductive-assisted method facilitates the selection of embryos without the hereditary cancer predisposition, offering confirmed carriers the opportunity to have a genetically-related unaffected child while avoiding pregnancy termination. Our study showed that although the majority of confirmed carriers had already had a fulfilled desire to have children before genetic testing, only 19% expressed that, upon reflection, they would have considered PGT as a reproductive option. Among at-risk carriers, the positive attitude toward PGT was remarkably low (10%), given that two-thirds of them intended to have children. This is in contrast to a condition such as FAP, where childhood-onset disease necessitates invasive surveillance from the age of 12 and early prophylactic colectomy between the ages of 15 and 25 [26, 27]. By comparison, effective and relatively non-invasive cancer surveillance programs are available to *CDKN2A* PV carriers [1, 3, 5]. As a result, the use of PGT may be less urgent for *CDKN2A* PV carriers compared to individuals with hereditary cancer syndromes that involve burdensome preventive measures and surveillance methods and. However, it is noteworthy that even in FAP, only 30% of patients expressed a positive attitude toward PGT. This suggests that factors other than disease severity and surveillance practices contribute to PGT attitudes [8]. Studies of other hereditary cancer syndromes such as PJS, von Hippel-Lindau syndrome (VHL), and Li-Fraumeni syndrome (LFS) found that 35–52% of the individuals expressed a positive attitude toward PGT [7, 8, 11].

Furthermore, two-thirds of participants had never heard or did not recall having been told about PGT before the study. While the provided information in this study might have offered a basic understanding, it was likely insufficient to form a well-informed opinion about PGT. This aligns with findings from previous studies on HBOC couples [23, 29]. In one study, over 40% of participants reported difficulty making reproductive decisions, and 70% expressed a need for additional support [23]. Similarly, another study found that, despite 77% of participants receiving genetic counseling in which reproductive options were discussed, most couples (85%) were not equipped to make an informed choice about their preferred reproductive options, including PGT [29].

### Strengths and limitations

A strength of this study is the large sample size of confirmed *CDKN2A* PV carriers, which allows for in-depth exploration of their attitudes on important issues. However, the sample size of at-risk carriers was relatively small. In our study, the proportion of at-risk carriers was 16% (39 of 247 participants), which is comparable to the proportion of 13% in similar studies of other rare hereditary cancer syndromes, including FAP, LFS, and VHL [8, 11]. In addition, previous literature has reported lower response rates in younger adults (aged 20–30 years) and males, which is consistent with our study [30].

As a result of the low response rate (23%) among at-risk carriers, non-response bias may have been introduced [31]. While demographic characteristics such as age and sex were comparable between participants and non-participants, the extent to which the cohort of respondents represents the overall population of at-risk carriers remains uncertain.

Furthermore, our study only focused on the perspectives of confirmed and at-risk carriers. To gain a more comprehensive understanding of reproductive decision-making, including PGT, it would be valuable to also consider partners’ perspectives. Previous research with FAP patients and their partners has shown that while about two-thirds of couples have similar attitudes toward PGT, there can be discrepancies. In cases of disagreement, partners often have a more positive view of PGT (56%). Hence, including partner attitudes may provide a more nuanced insight into reproductive decision-making within couples affected by the *CDKN2A* PV.

Lastly, the generalizability of our findings may be limited by our homogenous study population. All participants were Dutch and participated in the LUMC’s cancer surveillance programs. Cultural attitudes and regulations regarding (prenatal) genetic testing, mortgages, and insurance can vary significantly across countries. Therefore, these findings might not be directly applicable to other international hereditary cancer cohorts.

## Conclusions

In summary, the most common motivations for genetic testing were to learn about personal and family cancer risk, and to intensify cancer surveillance practices. Most confirmed carriers underwent genetic testing after having children and were therefore unaware of their cancer risk when starting a family. Conversely, at-risk carriers in our study were all aware of their risk carrier status, but three-quarters also reported that their risk carrier status had minimal impact on their reproductive choices. Additionally, PGT was only considered by a small minority of participants. Understanding these attitudes can help health care providers to navigate the complexities surrounding these issues, especially for younger individuals facing difficult decisions about the timing of genetic testing, family planning, and the potential use of assisted reproductive options such as PGT.

### Electronic supplementary material

Below is the link to the electronic supplementary material.

Supplementary Material 1

Supplementary Material 2

## Data Availability

No datasets were generated or analysed during the current study.

## References

[1] Klatte DCF, Boekestijn B, Wasser M (2022). Pancreatic Cancer surveillance in carriers of a germline CDKN2A pathogenic variant: yield and outcomes of a 20-Year prospective Follow-Up. J Clin Oncol Oct.

[2] Gruis NA, van der Velden PA, Sandkuijl LA (1995). Homozygotes for CDKN2 (p16) germline mutation in Dutch familial melanoma kindreds. Nat Genet.

[3] Vasen H, Ibrahim I, Ponce CG (2016). Benefit of Surveillance for Pancreatic Cancer in High-Risk individuals: outcome of long-term prospective Follow-Up studies from three European Expert centers. J Clin Oncol Jun.

[4] Bishop DT, Demenais F, Goldstein AM (2002). Geographical variation in the penetrance of CDKN2A mutations for melanoma. J Natl Cancer Inst Jun.

[5] Halk AB, Potjer TP, Kukutsch NA, Vasen HFA, Hes FJ, van Doorn R (2019). Surveillance for familial melanoma: recommendations from a national centre of expertise. Br J Dermatol.

[6] Klatte DCF, Onnekink AM, Hinnen C et al (2023) Psychosocial issues of individuals undergoing surveillance for increased risk of melanoma and pancreatic cancer due to a germline CDKN2A variant: a focus group study. J Genet Couns Oct 25. 10.1002/jgc4.1820 10.1002/jgc4.1820 37876362

[7] van Lier MG, Korsse SE, Mathus-Vliegen EM (2012). Peutz-Jeghers syndrome and family planning: the attitude towards prenatal diagnosis and pre-implantation genetic diagnosis. Eur J Hum Genet Feb.

[8] Douma KF, Aaronson NK, Vasen HF, Verhoef S, Gundy CM, Bleiker EM (2010). Attitudes toward genetic testing in childhood and reproductive decision-making for familial adenomatous polyposis. Eur J Hum Genet Feb.

[9] PGT Netherlands - (2024) Annual Report of Preimplantation Genetic Testing in the Netherlands

[10] Quinn GP, Pal T, Murphy D, Vadaparampil ST, Kumar A (2012). High-risk consumers’ perceptions of preimplantation genetic diagnosis for hereditary cancers: a systematic review and meta-analysis. Genet Med Feb.

[11] Lammens C, Bleiker E, Aaronson N (2009). Attitude towards pre-implantation genetic diagnosis for hereditary cancer. Fam Cancer.

[12] van der Rhee JI, de Snoo FA, Vasen HFA et al (2011) Effectiveness and causes for failure of surveillance of CDKN2A-mutated melanoma families. *Journal of the American Academy of Dermatology*. 2011;65(2):289–296. 10.1016/j.jaad.2010.06.067 10.1016/j.jaad.2010.06.067 PMC3138884 21570154

[13] Klatte DCF, Boekestijn B, Onnekink AM (2023). Surveillance for pancreatic Cancer in high-risk individuals leads to Improved outcomes: a propensity score-matched analysis. Gastroenterol Jun.

[14] Kasparian NA, Meiser B, Butow PN, Simpson JM, Mann GJ (2009). Genetic testing for melanoma risk: a prospective cohort study of uptake and outcomes among Australian families. Genet Med Apr.

[15] *Journal of Medical Genetics*. 2021;58(4):264. doi:10.1136/jmedgenet-2019-106562 10.1136/jmedgenet-2019-106562 PMC8005797 32482799

[16] Vasen HFA, Gruis NA, Frants RR, van der Velden PA, Hille ETM, Bergman W (2000) Risk of developing pancreatic cancer in families with familial atypical multiple mole melanoma associated with a specific 19 deletion of p16 (p16-Leiden). Int J Cancer 87(6):809–811. https://onlinelibrary.wiley.com/doi/abs/10.1002/1097-0215%2820000915%2987%3A6%3C809%3A%3AAID-IJC8%3E3.0.CO%3B2-U 10956390

[17] Potjer TP, van der Stoep N, Houwing-Duistermaat JJ (2015). Pancreatic cancer-associated gene polymorphisms in a nation-wide cohort of p16-Leiden germline mutation carriers; a case-control study. BMC Res Notes Jun.

[18] Presymptomatic G (2024) Testing and Insurances

[19] Insurances (2024) and Hereditary Diseases

[20] Levin T, Mæhle L (2017). Uptake of genetic counseling, genetic testing and surveillance in hereditary malignant melanoma (CDKN2A) in Norway. Fam Cancer.

[21] Bodd TL, Reichelt J, Heimdal K, Møller P (2003). Uptake of BRCA1 genetic testing in adult sisters and daughters of known mutation carriers in Norway. J Genet Couns.

[22] Derks-Smeets IAP, Gietel-Habets JJG, Tibben A (2014). Decision-making on preimplantation genetic diagnosis and prenatal diagnosis: a challenge for couples with hereditary breast and ovarian cancer. Hum Reprod.

[23] Gietel-Habets J, de Die‐Smulders C, Derks‐Smeets I (2018). Support needs of couples with hereditary breast and ovarian cancer during reproductive decision making. Psycho‐oncology.

[24] Dekeuwer C, Bateman S (2013). Much more than a gene: hereditary breast and ovarian cancer, reproductive choices and family life. Med Health Care Philos.

[25] Donnelly L, Watson M, Moynihan C (2013). Reproductive decision-making in young female carriers of a BRCA mutation. Hum Reprod.

[26] van Leerdam ME, Roos VH, van Hooft JE (2019). Endoscopic management of polyposis syndromes: European Society of Gastrointestinal Endoscopy (ESGE) Guideline. Endoscopy Sep.

[27] Lynch HT, Chapelle Adl (2003). Hereditary Colorectal Cancer. N Engl J Med.

[28] Genoff Garzon MC, Rubin LR, Lobel M, Stelling J, Pastore LM (2018). Review of patient decision-making factors and attitudes regarding preimplantation genetic diagnosis. Clin Genet.

[29] Reumkens K, Tummers MHE, Severijns Y (2021). Reproductive decision-making in the context of hereditary cancer: the effects of an online decision aid on informed decision-making. J Community Genet Jan.

[30] Hansen E, Fonager K, Freund KS, Lous J (2014) The impact of non-responders on health and lifestyle outcomes in an intervention study. *BMC Research Notes*. /09/11 2014;7(1):632. 10.1186/1756-0500-7-632 10.1186/1756-0500-7-632 PMC4175272 25213806

[31] Etter JF, Perneger TV (1997). Analysis of non-response bias in a mailed health survey. J Clin Epidemiol Oct.

