# Supplementary material for: Attitudes toward genetic testing, family planning and preimplantation genetic testing in families with a germline CDKN2A pathogenic variant
Source: Fam Cancer. 2024 Jun 1;23(3):255–65. doi: 10.1007/s10689-024-00401-3 (PMC11255069; doi:10.1007/s10689-024-00401-3)
Supplement: Supplementary file 1 — Supplementary Material 1 [file 10689_2024_401_MOESM1_ESM.docx]

**Supplementary table 1. Attitudes toward PGT**

|  | Confirmed carriers  (*n* = 208) | At-risk carriers  (*n* = 39) |
| --- | --- | --- |
| Have you heard of PGT before? ^a^  *No*  *Yes* | 134 (66%)  68 (34%) | 24 (62%)  15 (39%) |
| Would you have preferred to receive counseling about PGT? ^a^  *Yes*  *I don’t know*  *No* | 43 (21%)  64 (32%)  95 (47%) | 4 (10%)  15 (36%)  21 (54%) |
| Would you (have) consider(ed) using PGT for family planning? ^b^  *Yes*  *Maybe*  *No* | 38 (19%)  66 (33%)  95 (47%) | 4 (10%)  25 (64%)  10 (26%) |

PGT, preimplantation genetic testing; PV, pathogenic variant. ^a^ 6 confirmed carriers missing. ^b^ 9 confirmed carriers missing.
