# Supplementary material for: Attitudes toward genetic testing, family planning and preimplantation genetic testing in families with a germline CDKN2A pathogenic variant
Source: Fam Cancer. 2024 Jun 1;23(3):255–65. doi: 10.1007/s10689-024-00401-3 (PMC11255069; doi:10.1007/s10689-024-00401-3)
Supplement: Supplementary file 2 — Supplementary Material 2 [file 10689_2024_401_MOESM2_ESM.docx]

**General**

1. What is your sex?

□ Male

□ Female

□ Other

□ Rather don’t tell

2. What is your relationship status?

**□** Married

□ Living together

□ Having a relation, not living together

□ Single

□ Divorced

□ Widower

3. What is your highest education?

□ None

□ Primary school

□ Lower vocational school (*in Dutch* “lbo / lts”)

□ Intermediate vocational school (*in Dutch* “mbo / mts”)

□ Lower secondary school (*in Dutch* “mavo / vmbo”)

□ Higher secondary school (*in Dutch “*hbs / havo / vwo”)

□ College (*in Dutch “*hbo / hts”)

□ University

4. Do you have children?

□ Yes → Continue to question 5

□ No → Continue to question 6

5. How many children do you have and what is their age?

**Fill in:**

| **Son / daughter** | **Age** |
| --- | --- |
| 1. |  |
| 2. |  |
| 3. |  |
| 4. |  |
| 5. |  |
| 6. |  |

6a. Do you work?

□ Yes, I have a paid job

□ Yes, unpaid work (e.g. volunteer work)

□ No → Continue to question 6b

6b. You don’t work. What situation applies best?

□ I have retired

□ I can’t work because of health issues

□ Other ________________________________________

7a. Do you smoke?

□ Yes, ____ cigarettes per day → proceed to question 7b

□ No, but I used to in the past → proceed to question 7c

□ No, never smoked → proceed to question 8

7b. If you still smoke, do you plan to reduce or quit?

□ Yes, I am already in the process of reducing

□ Yes, sometime within the next 6 months

□ Yes, sometime in the future (more than 6 months)

□ No, I do not plan to do so (for now) → proceed to question 8

7c. You have quit or are planning to quit smoking. Did the *CDKN2A* pathogenic variant play a role in the decision to quit smoking?

□ Yes

□ No

7d. When did you quit smoking and for how long did you smoke?

- I quit ________ years ago.
- I smoked for ________ years, approximately ________ cigarettes per day.

8. Do you drink alcohol?

□ Yes → proceed to question 9

□ No → proceed to question 10

9a. What type of alcohol do you primarily drink? Check one answer.

□ Beer

□ Wine

□ Spirits

9b. How many glasses of alcohol do you (approximately) drink per week?

□ 1 – 7 glasses

□ 8 – 20 glasses

□ 21 – 40 glasses

□ More than 40 glasses

10. What is your height? _________________ (cm)

11. What is your weight? ________________ kg

**Medical background on cancer**

| The *CDKN2A* pathogenic variant increases the risk of cancer. Particularly, the risk of developing melanoma (70%) and pancreatic cancer (15-20%) is elevated. We would like to know if you or your family members have experienced this. |
| --- |

8. Have you had cancer? You can select multiple answers.

□ No → proceed to question 12

□ Yes, melanoma → fill in questions 9a and 9b

□ Yes, pancreatic cancer → fill in question 10

□ Yes, another type of cancer → fill in question 11

The following questions apply if you have had melanoma.

9a. How many times have you had melanoma?

□ Once

□ 2 – 5 times

□ 6 – 10 times

□ More than 10 times

9b. How old were you when melanoma was first diagnosed in you?

Age: ___________________

The following question applies if you have had pancreatic cancer.

10. How old were you when pancreatic cancer was diagnosed in you?

Age: ___________________

The following question applies if you have had another type of cancer.

11. In case you have experienced another type of cancer, what type of cancer was it? And how old were you at that time? Note as in the example below.

**Fill in:**

| **Type of cancer** | **Age at diagnosis** |
| --- | --- |
| 1. |  |
| 2. |  |
| 3. |  |
| 4. |  |
| 5. |  |
| 6. |  |

**Cancer within the family**

| The following questions help us determine who in your family has had cancer. First-degree relatives are your parents, siblings and children. |
| --- |

12a. Do you have first-degree family members who have had melanoma?

□ Yes

□ No → proceed to question 13

12b. At what age were first-degree relatives diagnosed with melanoma? Fill in as in the example below.

**Example**

| ***Melanoma*** | ***First-degree family member*** | ***Age at diagnosis*** |
| --- | --- | --- |
|  | *1. Father* | *42 years* |
|  | *2. Sister* | *29 years* |
|  | *3. Etc.* |  |

**Fill in:**

| **Melanoma** | **First-degree family member** | **Age at diagnosis** |
| --- | --- | --- |
|  | 1. |  |
|  | 2. |  |
|  | 3. |  |
|  | 4. |  |
|  | 5. |  |
|  | 6. |  |
|  | 7. |  |
|  | 8. |  |
|  | 9. |  |
|  | 10. |  |

13a. Do you have first-degree family members who have had pancreatic cancer?

□ Yes

□ No → proceed to question 14

13b. At what age were first-degree relatives diagnosed with pancreatic cancer?

**Fill in:**

| **Pancreatic cancer** | **First-degree family member** | **Age at diagnosis** |
| --- | --- | --- |
|  | 1. |  |
|  | 2. |  |
|  | 3. |  |
|  | 4. |  |
|  | 5. |  |
|  | 6. |  |
|  | 7. |  |
|  | 8. |  |
|  | 9. |  |
|  | 10. |  |

14a. Do you have first-degree family members who have had another type of cancer?

□ Yes

□ No → proceed to question 15

**Fill in:**

| **Other type of cancer** | **First-degree family member** | **Age at diagnosis** |
| --- | --- | --- |
|  | 1. |  |
|  | 2. |  |
|  | 3. |  |
|  | 4. |  |
|  | 5. |  |
|  | 6. |  |
|  | 7. |  |
|  | 8. |  |
|  | 9. |  |
|  | 10. |  |

Do you have any additional comments or remarks about your (family) history with cancer?

__________________________________________________________________________________

__________________________________________________________________________________

__________________________________________________________________________________

***CDKN2A* pathogenic variant inheritance and genetic testing**

| The *CDKN2A* pathogenic variant is heritable and therefore often occurs in more than one person in a family. Children of individuals with the *CDKN2A* pathogenic variant have a 50% likelihood of carrying the pathogenic variant. Genetic testing can determine whether someone carries the pathogenic variant through laboratory analysis. |
| --- |

15. How old were you when you realized you could have inherited the *CDKN2A* pathogenic variant (before any genetic testing was done)?

Age: ______________________ years

We are interested in your opinion on genetic testing, even if you have not yet undergone it. If the question does not directly apply to you, please still select the most appropriate answer.

16a. Have you undergone genetic testing yourself?

□ Yes → proceed to question 16b

□ No → proceed to question 16c

16b. Was the *CDKN2A* pathogenic variant detected in the genetic testing?

□ Yes, the *CDKN2A* pathogenic variant was detected

□ No, the *CDKN2A* pathogenic variant was not detected

16c. You have not undergone genetic testing (yet). Do you intend to undergo genetic testing in the future?

□ Yes, I intend to undergo genetic testing at a later time → Proceed to question 20

□ I’m unsure whether I want to undergo genetic testing → Proceed to question 20

□ No, I don’t want to undergo genetic testing → Proceed to question 20

17. How old were you when you underwent genetic testing?

Age: ______________________ years

18. You have undergone genetic testing or plan to do so. What are the main reasons for you to undergo this testing?

**Check up to 3 answers**:

□ To become eligible for pancreatic cancer surveillance and/or intensify skin surveillance

□ To gain more certainty about my risk of melanoma and pancreatic cancer

□ For my children

□ To feel a greater sense of control

□ For family planning decisions

□ General future planning (other than family planning, such as work or relationships)

□ A healthcare professional has referred me

□ Requested by a family member

□ To contribute to scientific research

□ Other, namely _____________________________________________________________

19. Have you postponed genetic testing because of wanting to secure a mortgage or insurance?

□ Yes

□ No

□ I don’t know

20. You have not undergone genetic testing (yet). What are the main reasons for you not to do so?

**Check up to 3 answers**:

□ I’m still too young

□ I already undergo skin checks and therefore feel that genetic testing is not necessary

□ The genetic test result may have financial implications for obtaining a mortgage/insurance

□ I don't want the results of genetic testing to affect my daily life

□ The test will increase my fear of cancer

□ I don't want the genetic test result to influence my family planning decisions

□ Testing was discouraged by my close friends or family members

□ I find it to be confronting to undergo testing

□ Other, namely _____________________________________________________________

Do you have any additional comments or remarks about your about the decision to undergo/not undergo genetic testing?

__________________________________________________________________________________

__________________________________________________________________________________

__________________________________________________________________________________

**Family planning**

| We would like to know whether the *CDKN2A* pathogenic variant has played a role in your decision to have children, or whether it might play a role in the future. Even if you do not have children, we are interested in your point of view. |
| --- |

We would like to know if the *CDKN2A* pathogenic variant has played a role in the decision to have children or may play a role in the future.

21. Do you currently have a desire to have children?

□ Yes

□ No, my desire to have children has already been fulfilled

□ No, I don’t have a desire to have children

□ I don't know

22. Has the (potential) *CDKN2A* pathogenic variant influenced your desire to have children?

□ No → Go to question 23

□ Somewhat → Go to question 24

□ Yes → Go to question 24

23. For what reasons has the *CDKN2A* pathogenic variant not influenced your desire to have children? **You can select multiple answers. Because...**

□ ... I didn’t know about the presence of the *CDKN2A* pathogenic variant in my family when starting a family

□ ... I had not yet undergone genetic testing (before having children)

□ ... The *CDKN2A* pathogenic variant does not necessarily have to result in serious health consequences

□ ... I believe that the chances that I will get sick in the future are low

□ … I believe that the chances that my children will get sick in the future are low

□ ... Of religious considerations

□ Other, namely _____________________________________________________________

24. For what reasons has the *CDKN2A* pathogenic variant influenced your desire to have children (somewhat)? **You can select multiple answers. Because...**

□ ... I am currently ill or expect to become ill in the future

□ ... I am afraid/was afraid that my child could be a carrier

□ ... I find it difficult that one child could potentially be a carrier and the other not

□ ... my partner is/was afraid that our child could be a carrier

| **Preimplantation Genetic Testing (PGT)**  PGT is a reproductive method of screening embryos for a genetic condition to reduce the chance of transferring the condition. PGT is an option for people with a *CDKN2A* pathogenic variant who want to have children. Using IVF (in vitro fertilization), eggs are fertilized with sperm outside the body. These embryos are then genetically tested for the presence or absence of the *CDKN2A* pathogenic variant. The embryo without the *CDKN2A* pathogenic variant is then implanted into the uterus to continue the pregnancy. We are interested in your opinion about this reproductive method. |
| --- |

25. Have you heard of PGT before this questionnaire?

□ Yes

□ No

26. Would you have preferred to have received more information about PGT?

□ Yes

□ No

□ I don't know

27. Would you consider undergoing an IVF process with PGT yourself?

□ Yes, this would definitely be an option for me

□ This might maybe be an option for me

□ No, this would definitely not be an option for me

**Children and genetic testing**

The following questions are about children and genetic testing for the *CDKN2A* pathogenic variant. We are interested in your experience with or opinion about pursuing genetic testing for your (potential) children.

If you do not have children, you can skip all following questions.

28. Have you told your children that you are a (potentially) carrier of the *CDKN2A* pathogenic variant or have an increased risk of cancer?

□ Yes

□ No

29. Have you told your child(ren) that they can have inherited the *CDKN2A* pathogenic variant?

□ Yes

□ No → proceed to question 32

30. Your children are aware of the risk of the *CDKN2A* pathogenic variant, at what point was this discussed?

□ After I was diagnosed with the pathogenic variant

□ It came up naturally over the years

□ In response to questions from my child(ren)

□ When my children were old enough

□ In response to my own or another family member's illness

□ At another time, namely ___________________________________________________________

31. How old were your children approximately when the risk of the *CDKN2A* pathogenic variant was discussed?

| **Child**  (circle which one is applicable) | **Age** |
| --- | --- |
| 1: son / daughter |  |
| 2: son / daughter |  |
| 3: son / daughter |  |
| 4: son / daughter |  |
| 5: son / daughter |  |

32. Your children are not aware of the possible *CDKN2A* pathogenic variant risk, what is the main reason for not telling them?

**Check up to 2 answers:**

□ Because I don't know (yet) if I have the *CDKN2A* pathogenic variant

□ I'm afraid to tell my child(ren)

□ I think my child(ren) are still too young

□ It is not important for my child yet because he/she is already under skin surveillance

□ Other, namely _____________________________________________________________

33. Would you recommend your children to undergo genetic testing?

□ Definitely yes

□ Probably yes

□ Maybe

□ Definitely not

34. If you suppose that your child wants to undergo genetic testing. What age would you recommend to pursue genetic testing?

□ Between 12 – 17 years

□ Between 18 – 25 years

□ Between 26 - 35 years

□ Between 36 – 45 years

□ Other, _____________________________________________________________________

35. Can you explain why you think this is the most suitable age for genetic testing?

__________________________________________________________________________________

__________________________________________________________________________________

__________________________________________________________________________________

__________________________________________________________________________________
